# The association of dietary components with depression and anxiety symptoms: findings from a cross-sectional survey

**DOI:** 10.3389/fnut.2025.1546564

**Published:** 2025-06-04

**Authors:** Kerri M. Gillespie, Eva Kemps, Melanie J. White, Selena E. Bartlett

**Affiliations:** ^1^School of Clinical Sciences, Queensland University of Technology, Brisbane, QLD, Australia; ^2^Institute of Mental Health and Wellbeing, College of Education, Psychology and Social Work, Flinders University, Adelaide, SA, Australia; ^3^School of Psychology and Counselling, Queensland University of Technology, Brisbane, QLD, Australia

**Keywords:** sugar-sweetened beverages, SSB, caffeine, dietary fibre, depression, anxiety

## Abstract

**Introduction:**

It is understood that diet has a significant impact on health. However, the associations between individual macronutrients and mental health are poorly understood. We aimed to investigate the relationship between dietary components and symptoms of depression and anxiety.

**Methods:**

A cross-sectional study of 129 healthy adults was conducted using an online food frequency questionnaire and measures of depression, anxiety, and early life experiences.

**Results:**

Of the sample, 31% experienced moderate or severe anxiety and/or depression. Sugar-sweetened beverage (SSB) intake was positively associated with depression (*β* = 4.827; 95% CI: 0.954, 8.7; *p* = 0.015). Dietary fibre was negatively associated with anxiety (*β* = -2.306; 95% CI: -3.791, -0.82; *p* = 0.003). Moderate caffeine consumption (100-300mg) was associated with reduced depression (*β* = -4.099; 95% CI: -7.049, -1.15; *p* = 0.007). Women experienced higher rates of depression and anxiety. We found evidence suggesting an association between high-fructose corn syrup and depressive symptoms and a potential protective effect of fibre on anxiety. A U-shaped curve for caffeine may be present, with moderate consumption associated with improved mental health.

**Discussion:**

A significant positive association was found between SSB consumption and depression, while a negative association was found between fibre consumption and anxiety. Additional prospective studies with repeated dietary data capture are needed to affirm these findings.

## 1 Introduction

Depression and anxiety are highly comorbid psychological disorders that make significant contributions to global health-related burden (1). Despite improved treatments, the prevalence and incidence of both disorders have increased over the past decades (2–4). A great deal of evidence now exists to support diet as a modifiable risk factor for mental illness, particularly depression and anxiety. Studies investigating dietary patterns have found diets high in fruits, vegetables, whole grains, nuts, and fish to be associated with improved physical and mental health (5, 6). Conversely, diets high in sugars, fats, and salt have been associated with mental illness, chronic disease, and cognitive decline (7–9). While whole-of-diet studies have highlighted the benefits of these dietary regimens, the specific beneficial or detrimental effects of individual components of these diets are not as well understood.

Two macronutrients most consistently associated with mental health are sugar and fibre. One of the major theories behind the impacts of these dietary components relates to their influence on gut microbiome. Sugar consumption can alter gut microbial diversity, impairing gut lining and leading to systemic inflammation and neuroinflammation, thought to be the cause of mental sequelae (10, 11). Mice that chronically consume high sucrose diets display significant alterations in neurological structure and function, including altered hippocampal neurogenesis (12). Human studies into sugar consumption have been inconsistent, but a number of large-scale studies have found that frequent consumption of sugar-sweetened beverages (SSBs) increased the risk of depression and suicide ideation (13–15). Other research indicates a potential negative impact of excessive sugar intake on anxiety (16). The World Health Organisation recommends that adults consume no more than 10% of their total energy intake as sugars, or limit intake to roughly 12 teaspoons (50 grams) per day (17). Recent census data from Australia reports that average daily consumption exceeds this amount (66.9 grams and 61.6 grams of free and added sugars per person per day, respectively, or approximately 12.3 and 11.3% of total dietary energy) (18).

More frequently identified in prior research is the relationship between fibre and mental health. Fermentation of dietary fibre in the distal gut leads to the formation of short-chain fatty acids, which are thought to have anti-inflammatory functions, improve gut lining integrity, and play a role in the regulation of lipids, cholesterol, and glucose metabolism (19). Increased dietary fibre intake has further been shown to restore healthy gut microbiota, and reduce symptoms of depression and anxiety (20). It is recommended that adults consume around 25 g of dietary fibre per day, or about 14 g for every 4,000 kilojoules (roughly 2.8% of total energy intake) (21, 22). In 2022, only around 6% of Australian adults consumed the recommended daily amount of vegetables, and around 44% consumed the recommended amount of fruit (18). Apparent consumption of grains and cereals was also below the recommended daily intake for 67 and 76% of men and women, respectively (18). These figures suggest that Australians are unlikely to consume adequate amounts of dietary fibre. In support, a study conducted using the 2011–2012 Australian population data found that less than 20% of adults consumed the recommended amount of dietary fibre to reduce chronic disease risk (23). Consumption of fruits and vegetables, known for their high fibre content, has been linked to lower rates of depression, anxiety, and stress (24). One study identified an inverse relationship between dietary fibre and depression, but only in women (25). However, not all studies have identified these associations, and less evidence exists for the benefits of fibre supplements (26). The source of dietary fibre may also be a factor in the role of fibre on mental health outcomes (27).

Other dietary components have also been associated with mental health. Protein consumption in the Western world is up to double the recommended intake, consisting of predominantly animal protein (28). Studies investigating the impact of protein consumption on mood have found conflicting results. Protein intake has been linked to a reduction in depression and anxiety (29, 30), but may affect men and women differently (31, 32). Source of protein intake may also be a critical factor, with some studies finding higher stress and depression linked to animal protein compared to plant protein (32, 33), while another observed the reverse relationship (30).

Other dietary components that have had conflicting findings in terms of their relationship with mental health are caffeine and alcohol. Alcohol has traditionally been associated with increased depression and anxiety, but this may be related to the comorbid presence of alcohol use with depression and anxiety, rather than a causal relationship (34). Around one-third of the Australian population aged 14 years and over report drinking more than 10 standard drinks per week, or more than four standard drinks in a day at least once per month (35). Large longitudinal studies have identified greater odds of depression and/or anxiety, and higher symptom severity in non-drinkers (24, 36). Studies of coffee and caffeine consumption have been even more conflicted, from observing an inverse relationship between coffee and depression (37), to finding a significant increase in depression, anxiety, and stress with higher caffeine intake (38), to finding no relationship at all (39). These findings may be due to sex differences, as some researchers have observed associations in women but not men (40, 41). Close to half the Australian adult population (46%) reports drinking around 330 mL of coffee per day (42).

There is a general consensus regarding the benefits of broad, dietary patterns such as Mediterranean or Nordic diets (43). However, studies investigating the impacts of individual macronutrients and dietary components on depression and anxiety have had conflicting outcomes. The current study aimed to investigate and identify the possible beneficial or detrimental impacts of different dietary components on depression and anxiety, while controlling for sociodemographic factors known to impact mood.

## 2 Methods

### 2.1 Study design

The study was a cross-sectional design using online surveys.

### 2.2 Participants

Healthy participants aged 18 to 70 years of age were recruited via the networks of the researchers, social media, and university mailing lists. The study recruited 144 participants. Two participants were flagged as potential replicate responses by the online survey software and were removed. An additional 13 participants were excluded for not completing the online dietary questionnaires, leaving a total of 129 participants. Participants were predominantly highly educated, and female (see Table 1).

**Table 1:** Demographic characteristics and survey results.

Item	*N* (%)	Mean (SD)	Range
Age	129 (100)	37.3 (14.3)	18–70
Gender			
Female	93 (72.1)		
Male	36 (27.9)		
Highest educational qualification			
Grade 12	19 (14.7)		
Trade / technical / vocational training	8 (6.2)		
Undergraduate degree	44 (34.1)		
Postgraduate degree	58 (45.0)		
Household income			
Under $50,000	37 (28.7)		
$50,100–$110,000	30 (23.3)		
Over $110,000	62 (48.1)		
Smoking			
Never smoked	105 (81.4)		
Ex smoker	22 (17.1)		
Current smoker	2 (1.6)		
Ethnicity			
Caucasian	89 (69.0)		
Asian	33 (25.6)		
Aboriginal and/or Torres Strait Islander	2 (1.6)		
Other	5 (3.8)		
BMI		24.8 (4.7)	17.5–41.7
Early life experiences		35.6 (13.4)	15–69
Low (<33)	62 (48.1)		
High (≥33)	67 (51.9)		
Depression		53.1 (8.2)	37.2–74.2
Healthy	74 (57.4)		
Mild	26 (20.2)		
Moderate	27 (20.9)		
Severe	2 (1.6)		
Anxiety		54.2 (8.6)	37.1–77.2
Healthy	70 (54.3)		
Mild	27 (20.9)		
Moderate	30 (23.3)		
Severe	2 (1.6)		
Dietary fibre^a^		2.3 (0.9)	0.5–5.2
Total sugar^a^		16.5 (7.5)	1.4–37.8
Protein^a^		17.7 (6.6)	5.6–39.1
Caffeine (mg)		126.7 (144.8)	0–719.8
Less than 100 mg	72 (55.8)		
100-300 mg	43 (33.3)		
Over 300 mg	14 (10.9)		
Alcohol per week (standard drinks)		3.4 (7.8)	0–60
Less than 1 standard drink per week	54 (45.0)		
1 or more standard drinks per week	66 (55.0)		
Sugar sweetened beverages per week (250 mL cups)		3.7 (7.4)	0–49
Less than 7 cups per week	113 (87.6)		
7 cups or more per week	16 (12.4)		

a Presented as percentage of energy consumed.

### 2.3 Measures

#### 2.3.1 Anxiety

Anxiety was measured using the Patient-Reported Outcomes Measurement Information System (PROMIS): Emotional Distress – Anxiety - Short Form 8a (44). This eight-item survey asked participants how often they had experienced anxiety-related states (e.g., “I felt fearful”) over the past 7 days on a five-point Likert scale from never to always. Previous studies have found it demonstrated good internal consistency (Cronbach’s *α* = 0.93), with a single-factor solution accounting for 79% of total variance (45). Internal consistency for the present sample was also high (Cronbach’s α = 0.94).

#### 2.3.2 Depression

Depression was measured using the Patient-Reported Outcomes Measurement Information System (PROMIS): Emotional Distress – Depression – Short Form 8b is a similar, eight-item Likert-scale survey (44). Participants were asked to rate how often they experienced depression-related states (e.g., “I felt worthless”) on a scale of Never to Always. Previous studies using this tool have found it demonstrated good internal consistency (Cronbach’s *α* = 0.95), with a single-factor solution accounting for 85% of total variance (45). The present sample also showed high internal consistency (Cronbach’s *α* = 0.94).

#### 2.3.3 Dietary intake

Dietary intake was assessed using the free, web-based “Automated Self-Administered 24-h” (ASA24-Australia) dietary assessment tool.^1^ This measure is a widely used and validated tool based on the United States Department of Agriculture’s Automated Multiple-Pass Method and The Food Intake Recording Software System (46). This online site calculated macronutrient intake for one 24-h period. Participants choose an eating occasion (e.g., breakfast, lunch), before choosing foods and beverages from a list of food categories. Participants are then asked detailed questions relating to preparation and portions, with visual guides provided to assist with consistency in portion sizes. Additional questions prompt participants to recall any food intake they may have overlooked, reducing the risk of recall bias.

#### 2.3.4 Beverage intake

Beverage intake for alcohol and SSBs was collected from an online demographic survey. Participants were asked to rate their frequency of beverage consumption on a scale of ‘never or rarely’; ‘more than once a month but less than once a week’; ‘once or twice a week’; ‘most days’; or ‘every day’. Participants were then asked how many standard drinks of alcohol, or 250 mL cups of SSBs, they usually consumed on these occasions. Examples of a standard drink for different beverage types was given. Beverage dose was estimated from these responses.

#### 2.3.5 The early life experiences scale (ELES)

The early life experiences scale (ELES) (47) is a 15-item survey that examines perceptions of childhood. Participants are asked to rate statements relating to their parents and home life on a scale of completely untrue (1) to very true (5). Scores are summed to create three subscales: feeling Unvalued; Submissiveness; and feeling Threatened. A total score was also calculated, and a cutoff of >32 used (48).

### 2.4 Covariates

Variables that were included in the preliminary analyses were: dietary fibre, total sugar, protein, caffeine, alcohol consumed per week (in standard drinks), sugar-sweetened beverages consumed per week (in 250 mL cups), BMI, and early life experiences (ELES total score). Fibre, total sugar, and protein were calculated as a percentage of total energy intake. Early life experiences have been shown to be associated with later life depression and anxiety (47), and were therefore controlled for in the final analysis. Based on the literature, age, gender, and income were also included in the model to control for these potentially confounding variables. Education was strongly associated with age in the current sample, and therefore income was chosen as the sole indicator of socioeconomic status. Smoking was not controlled for due to the low number of current smokers. All carbohydrates were grouped together in the final dataset and were therefore excluded from the analysis. Simple carbohydrates have been associated with poor mental health, whereas complex carbohydrates are more frequently associated with improved physical and mental health, and the inclusion of these two types together would have provided invalid results (16).

### 2.5 Power analysis

A power calculation was conducted using G*Power software for linear multiple regression (fixed model, R2 deviation from zero). To detect a medium effect size (f2 = 0.15) with 80% power, alpha error probability of *α* = 0.05, and six predictors, a minimum sample size of 98 was required.

### 2.6 Statistical analysis

Descriptive and inferential statistics were performed using SPSS version 29.0. All covariates, aside from total sugar, presented non-normal distributions according to the Kolmogorov–Smirnov normality test. Correlations were therefore conducted using the non-parametric Spearman’s Rho correlation coefficient. Spearman’s Rho correlations and multivariate linear regression models were conducted to determine the impact of the independent variables on depression and anxiety. Variables found to be significantly related to depression or anxiety were included in the final models, while also controlling for age, sex, and socioeconomic status.

### 2.7 Ethical statement

The study protocol was approved by the Queensland University of Technology Human Research Ethics Committee (Ethics ID: 5872). All participants provided written, informed consent prior to taking part in the study.

## 3 Results

### 3.1 Participant characteristics

Over half of the respondents reported high levels of negative early life experiences (see Table 1). When combined, 40 (31%) respondents experienced moderate or severe anxiety and/or depression. The majority of participants (74.4%) reported that they had consumed their usual daily amount of food the day they entered their dietary data, and a small amount reported to have eaten less (15.5%) or more (10.1%) than usual. The majority of respondents (75.2%) consumed less than the recommended 2.8% of total energy as fibre, and 78.3% consumed more than 10% of their total daily energy as sugar. Recommended daily protein is around 10 to 35% of daily energy (49). Only 9.3% of respondents consumed less than these daily protein recommendations, and one consumed more than 35% of total energy as protein. Only 4.7% of respondents consumed more than the recommended maximum amount of 400 mg per day of caffeine (50). For alcohol, 20.8% of respondents had not consumed alcohol in the past week, and 9.2% had consumed more than the recommended maximum amount of 10 drinks per week (51). Of the 85.3% of respondents who reported consuming sugar-sweetened beverages, 48.1% drank at least weekly, and 12.4% drank 7 or more cups per week.

### 3.2 Correlations

Sugar-sweetened beverage consumption per week was significantly positively associated with both depression and anxiety (see Table 2). Fibre and caffeine intake were both negatively associated with anxiety. In addition, drinking more SSBs was associated with consuming less fibre. Depression and anxiety scores were lower with increasing age. Older participants also appeared to drink fewer SSB, but more alcohol and caffeine.

**Table 2:** Correlations between continuous study variables.

Items	1	2	3	4	5	6	7	8	9	10
1.Fibre	–									
2.Total sugar	0.11	–								
3.Protein	−0.22*	−0.45**	–							
4.Total Energy	−0.2*	−0.11	−0.11	–						
5.SSB	−0.36***	−0.01	0.08	0.19*	–					
6.Alcohol	−0.09	−0.2*	0.04	−0.09	−0.13	–				
7.Caffeine	0.08	0.21*	−0.17	0.09	−0.09	0.24**	–			
8.BMI	−0.07	−0.2*	0.06	0.08	0.13	0.09	−0.08	–		
9.Age	0.17	−0.03	−0.06	−0.11	−0.18*	0.32***	0.27**	0.23**	–	
10.Early life experiences	−0.12	0.03	−0.12	0.13	−0.01	−0.04	0.02	−0.04	−0.05	–
11.Depression	−0.07	−0.02	−0.13	−0.1	0.18*	−0.13	−0.18*	0	−0.18*	0.39***
12.Anxiety	−0.22*	−0.01	−0.11	0.04	0.2*	−0.08	−0.28***	−0.1	−0.27**	0.36***

* *p* < 0.05, ** *p* < 0.01, ***p < 0.001.

### 3.3 Linear regression models

Bivariate linear regression analyses were conducted to determine which covariates were significantly related to depression or anxiety (see Tables 3, 4). Significant variables were entered into the final linear regression models with gender, age, and income. Tables 3, 4 present unstandardised beta coefficients. These describe the change in the dependent variable (depression or anxiety) for every 1 unit increase in an independent continuous variable, or when compared with the reference category in a categorical variable. Experiencing adverse early life experiences was associated with higher depression and anxiety. Higher incomes were associated with lower depression and anxiety. A U-shaped curve was observed in caffeine intake, with moderate doses of caffeine (100-300 mg) associated with significantly lower depression and anxiety. Consumption of at least seven cups of SSBs per week was associated with higher depression, but not anxiety. Fibre intake was seen to be inversely associated with anxiety but showed no relationship to depression. Age was also inversely associated with anxiety only.

**Table 3:** Bivariate and multivariable linear regression models to investigate correlates of depression.

Items	Bivariate linear regression		Multivariable model	
	*β* (95% CI)	*p*-value	*β* (95% CI)	*p*-value
Early life experiences				
Under 33	1.0 (Ref)	(Ref)	1.0 (Ref)	(Ref)
33 and over	5.730 (3.023, 8.436)	<0.001	4.695 (2.093, 7.298)	<0.001
**Age**	−0.083 (−0.183, 0.018)	0.105	−0.028 (−0.124, 0.068)	0.567
Gender				
Male	1.0 (Ref)	(Ref)	1.0 (Ref)	(Ref)
Female	2.739 (−0.441, 5.919)	0.091	3.692 (0.774, 6.610)	0.014
Income				
Less than $50, 000	1.0 (Ref)	(Ref)	1.0 (Ref)	(Ref)
$50, 100 to $110, 000	−1.793 (−5.677, 2.090)	0.363	−1.482 (−5.159, 2.196)	0.626
Over $110, 000	−5.176 (−8.459, −1.892)	0.002	−4.138 (−7.207, −1.069)	0.009
Sugar-sweetened beverages consumed per week (250 mL cups)				
Less than 7 cups per week	1.0 (Ref)	(Ref)	1.0 (Ref)	(Ref)
7 cups or more per week	3.266 (0.432, 6.101)	0.024	4.827 (0.954, 8.700)	0.015
Caffeine				
Less than 100 mg	1.0 (Ref)	(Ref)	1.0 (Ref)	(Ref)
100-300 mg	−3.914 (−7.008, −0.819)	0.014	−4.099 (−7.049, −1.150)	0.007
Over 300 mg	−1.571 (−6.261, 3.119)	0.509	−1.507 (−5.727, 2.713)	0.481
**Fibre**	−1.067 (−2.616, 0.481)	0.175		
**Total sugar**	0.000 (−0.193, 0.193)	0.998		
**Protein**	−0.135 (−0.352, 0.082)	0.221		
Alcohol				
Less than 1 standard drink per week	1.0 (Ref)	(Ref)		
1 or more standard drinks per week	−2.603 (−5.525, 0.320)	0.080		
**Body mass index**	0.101 (−0.208, 0.410)	0.520		

*β*, Unstandardised beta coefficient; CI, Confidence Interval; Ref, Reference category.

**Table 4:** Bivariate and multivariable linear regression models to investigate correlates of anxiety.

Items	Bivariate linear regression		Multivariable model	
	*β* (95% CI)	*p*-value	*β* (95% CI)	*p*-value
Early life experiences				
Under 33	1.0 (Ref)	(Ref)	1.0 (Ref)	(Ref)
33 and over	5.561 (2.727, 8.396)	<0.001	4.008 (1.290, 6.726)	0.004
**Age**	−0.156 (−0.257, −0.054)	0.003	−0.072 (−0.174, 0.030)	0.162
Gender				
Male	1.0 (Ref)	(Ref)	1.0 (Ref)	(Ref)
Female	1.369 (−1.962, 4.699)	0.418	3.166 (0.077, 6.254)	0.045
Income				
Less than $50, 000	1.0 (Ref)	(Ref)	1.0 (Ref)	(Ref)
$50, 100 to $110, 000	−2.953 (−7.026, 1.119)	0.154	−2.494 (−6.385, 1.398)	0.207
Over $110, 000	−4.850 (−8.293, −1.406)	0.006	−4.133 (−7.429, −0.836)	0.014
Caffeine				
Less than 100 mg	1.0 (Ref)	(Ref)	1.0 (Ref)	(Ref)
100-300 mg	−3.503 (−6.723, −0.283)	0.033	−2.204 (−5.323, 0.915)	0.164
Over 300 mg	−3.774 (−8.654, 1.106)	0.128	−3.513 (−7.975, 0.950)	0.122
**Fibre**	−2.410 (−3.973, −0.847)	0.003	−2.306 (−3.791, −0.820)	0.003
Sugar-sweetened beverages consumed per week (250 mL cups)				
Less than 7 cups per week	1.0 (Ref)	(Ref)		
7 cups or more per week	4.073 (−0.414, 8.561)	0.075		
**Total sugar**	0.009 (−0.191, 0.209)	0.929		
**Protein**	−0.084 (−0.310, 0.142)	0.465		
Alcohol				
Less than 1 standard drink per week	1.0 (Ref)	(Ref)		
1 or more standard drinks per week	−1.654 (−4.739, 1.431)	0.291		
**Body mass index**	−0.070 (−0.391, 0.251)	0.666		

*β*, Unstandardised beta coefficient; CI, Confidence Interval; Ref, Reference category.

After inclusion in the multivariable model, adverse early life experiences were still associated with higher depression (see Table 2). Earning over $110,000 and moderate caffeine consumption remained inversely associated with depression. Drinking 7 or more SSBs per week was associated with an almost 5-point increase in depression scores. Gender became significant, and being female was now also associated with higher depression scores.

When controlling for other variables, adverse early life experiences were still associated with higher anxiety. Being female also became significantly associated with higher anxiety in the multivariable model. Fibre remained significantly inversely associated with anxiety. However, the relationship between caffeine consumption and anxiety was no longer evident and age was no longer associated with any outcome.

## 4 Discussion

The study aimed to identify the impact of select macronutrients and dietary components on anxiety and depression in a sample of healthy adults. Importantly, the analyses examined the effects of these variables when considered together within the same model, and after controlling for important demographic characteristics. Participants reported relatively high rates of moderate and severe anxiety (24.9%) and depression (22.5%) compared to Australian population estimates of anxiety (17%) (52) or global estimates of anxiety (4%) (53) and depression (5%) (54). Consumption of food types was similar to that observed in recent Australian population data. However, there were far fewer participants considered to be consuming alcohol at a high-risk level compared to Australian population estimates.

SSBs, dietary fibre, and caffeine were the only dietary components associated with mood. Drinking seven or more cups of SSBs per week was associated with an almost five times higher risk of depression. This relationship has also been identified in several previous observational studies (55–57). The present study also supports previous literature that has identified relationships between fibre and anxiety (24), finding that anxiety scores decreased by 2.3 for every additional percentage of energy consumed as fibre. The study found no association between fibre consumption and depression. This could potentially be due to the role of physical activity, which has been linked to reductions in depression, as well as improvements in gut microbial diversity (58–60). It is also important to note that dietary information was only captured for one 24-h time period for each participant. Approximately 25% of the current sample reported consuming more or less than their normal diet on this single-day snapshot, though by how much their diet differed is unknown. Since the study utilised a cross-sectional design, directionality of effect cannot be established, and the chance of reverse causality must be acknowledged. There is some evidence that the presence of depression or anxiety can impact dietary behaviours, leading to increased consumption of unhealthy foods and reduced consumption of healthy foods (61, 62). Low mood has also been seen to impact motivation and activity levels, leading to reduced physical activity and further exacerbating poor mental health (63, 64).

No association was found between total dietary sugars, protein, or alcohol. This may be because the majority of the sample consumed a diet that was low in alcohol and caffeine, and high in protein and fibre. Few participants drank alcohol, and only a very small number drank at excessive levels. Participants also had a relatively healthy diet, high in fibre and protein. The relationships between protein and mental health could also be complicated by the source of the protein (animal or plant) (32, 33). The only potentially detrimental factor that was consumed excessively by the sample overall was sugar. Although no relationship between total dietary sugars and mood was found, this may be because a majority of added sugars are generally consumed as sugar-sweetened beverages. It has been recognised in prior research that the high-fructose corn syrups found in SSBs may be more detrimental to neurological function and mental health than other sugar types (8). Total sugars may include natural sources of sugar such as fruits and juices which may have different impacts on neurological function (11).

Caffeine consumption was associated with a reduction in depression at moderate levels. However, consumption of 300 mg or more (approximately three cups of coffee) showed a similar association to no caffeine (58–60). This conflicts with some prior research, associating caffeine consumption with increased anxiety and depression (38). A meta-analysis conducted by Wang and colleagues (37) identified an inverse relationship between coffee consumption and depression. However, their dose–response analysis of caffeine and depression identified a non-linear relationship, with significant reductions in depression risk at caffeine doses above 68 mg/day and below 509 mg/day. These findings suggest that other components within coffee may influence the effect of caffeine on mood. Individual physical or psychiatric factors are also likely to have an impact on the relationship between caffeine and anxiety or depression. The current study found that consuming more than 300 mg of caffeine per day was associated with increases in anxiety. A meta-analysis of caffeine and anxiety studies found that those with panic disorder were more vulnerable to caffeine-induced anxiety and panic attacks compared to healthy controls (65). The potential for reverse causality must also be considered here. Increases in depression or anxiety may lead to an increase or decrease in the amount of coffee an individual consumes. Smoking is also known to increase caffeine metabolism, which can reduce its efficacy and lead to increased caffeine consumption (66). Further research should investigate whether small amounts of caffeine may be beneficial for anxiety and/or depression while controlling for potential confounding factors such as psychiatric health and smoking status. Depression and anxiety are considered significant public health concerns that pose a substantial social and economic burden on individuals, families, and health systems (67). The global burden of depression and anxiety has risen considerably over the past three decades and has been further exacerbated by the COVID-19 pandemic (67–69). These conditions can impact physical health and are associated with worse subjective quality of life and a significantly higher risk of suicide (70, 71). An extensive body of research has identified associations between diet quality and mental health (72). Further investigation into the directionality and mechanisms of these relationships could provide cheaper, more accessible, non-invasive, and safer alternatives to pharmaceutical and psychiatric treatments. This information is imperative to advise on policies such as taxation on SSBs and other processed foods, and to inform regulations regarding food formulations to limit the amounts of harmful dietary components (such as added sugars, sodium, and saturated fats), which could improve population physical and mental health.

## 5 Strengths and limitations

The study has a number of notable strengths as compared to previous similar studies. In particular, the analyses controlled for demographics such as age, sex, and socioeconomic status. In addition, the study design included several dietary components to identify individual beneficial or detrimental impacts on mood. However, it did not collect in-depth data on physical activity, which has also been linked to mood (58–60). Furthermore, a sample size calculation was conducted showing that the study was adequately powered. Notably, the final sample was highly educated relative to the general population. Hence, a larger and more diverse sample would have improved the generalisability of the findings. The inclusion of additional macronutrient components would also have allowed for comparisons of impact and to control for potentially confounding dietary and lifestyle behaviours.

As the study was cross-sectional in design, directionality of impact could not be determined. Another limitation was the method used to capture dietary data. The food diary was completed only once over a 24-h period. Food consumed on that particular day may not have been reflective of participants’ regular daily consumption. However, most participants indicated that their reported intake was similar to their usual daily intake. Although this method is common in diet-related studies (73), it is reliant on participant memory, and understanding of portion sizes may differ between respondents even when detailed instructions are provided. An average of multiple food frequency questionnaires completed over successive days would be a preferable way to capture this data. Another strategy proposed for dietary data collection is ecological momentary assessment (EMA); a method of repeated, real-time sampling (74).

Previous studies have identified differences between men and women regarding the impact of fibre (25, 26) and sugar (55) on mental health. Considering the higher rates of mental illness in women, an improved understanding of the mechanisms of the sex-specific impacts of diet would be valuable and may support more individualised approaches to mental health treatment. Unfortunately, the present study sample included too few males to undertake a stratified regression analysis to robustly investigate any differences between males and females in terms of the impacts of dietary components on depression or anxiety.

## 6 Conclusion

Results from the study support the notion that high-fructose corn syrup may be detrimental to mental health. Given the global popularity of sugar-sweetened beverages that contain high levels of high-fructose corn syrup, investigating the impact of sugar-sweetened beverages on mental and physical health in order to inform policy around food and beverage regulation should be a national priority. The findings also support previous studies that identified an inverse association between dietary fibre and anxiety. A U-shaped curve for caffeine indicates that a moderate amount of caffeine may be beneficial for mental health, while exceeding a specific threshold may lead to increased anxiety and/or depression.

## Data Availability

The raw data supporting the conclusions of this article will be made available by the authors upon reasonable request.

## Footnotes

^1^ https://epi.grants.cancer.gov/asa24
